# Topline results for the feasibility, added value and satisfaction of remote digital self‐assessment for mild cognitive impairment in primary and specialized routine care with the app neotivCare

**DOI:** 10.1002/alz.095582

**Published:** 2025-01-09

**Authors:** Emrah Düzel, Martin Griebe, Harriet Sommer, Michael Schoettler

**Affiliations:** ^1^ German Center for Neurodegenerative Diseases (DZNE), Magdeburg Germany; ^2^ Institute of Cognitive Neurology and Dementia Research (IKND), Otto von Guericke University, Magdeburg Germany; ^3^ Institute of Cognitive Neuroscience, University College London (UCL), London United Kingdom; ^4^ Department of Neurology, University Medical Center Medical Faculty Mannheim, Heidelberg, Baden‐Württemberg Germany; ^5^ Roche Pharma AG, Grenzach‐Wyhlen, Baden‐Württemberg Germany

## Abstract

**Background:**

The timely diagnosis of mild cognitive impairment (MCI) in Alzheimer’s disease is challenging in routine care due to the complexity and time burden of required cognitive assessments. New unsupervised digital remote assessment tools could adress this challenge.

**Method:**

The multicentric healthcare study “re.cogni.ze” evaluated usability, adherence and acceptance of remote digital assessment (neotivCare, installed on private smartphone) over a period of 22 months with 27 specialist physicians (neurologists and psychiatrists), 13 GPs and 3 memory clinics in Germany.

**Result:**

765 persons with subjectively perceived memory problems were recommended neotivCare by their doctors. 574 patients (75%) followed the recommendation (mean age 67 ‐ SD 10; 50.2% female). 573 (93%) used the app at home (one visual memory test per week over a period 12 weeks). 496 patients (93%) completed the assessment and discussed the results using the in‐app generated findings letter with their doctor. Of these, 368 patients also completed a separate (independent from the app) survey about usability, difficulty, worries, experienced added value etc. 40% of the patients fell below the prevalidated cut‐off for MCI in the app‐tests. Specialists reported a higher satisfaction with the app compared to standard paper‐pencil tests (5.2 versus 4.4 on a 7 point Likert scale). There was no such difference for GPs. 71% of all doctors found that the app easy to use. 80% saw an added value in using the app. 71.5% of the patients thought that the app was easy to use. 67.6% reported an added value over paper pencil tests. Using the app reduced experienced worries in 51% of the patients and left worries unchanged in 29%. The patients rated the duration of the app‐use and the self‐testing at home favourably (both 8.5 scores on a Likert scale 0 ‐10).

**Conclusion:**

The topline results of the re.cog.nize study show that remote digital self‐assessment in routine care is feasible and is accepted by health care providers and patients. The high adherence rates indicate that a timely assessment of MCI is possible with this type of digital technology.

